# Host shifts in economically significant fruit flies (Diptera: Tephritidae) with high degree of polyphagy

**DOI:** 10.1002/ece3.8135

**Published:** 2021-09-22

**Authors:** Jiayao He, Ke Chen, Fan Jiang, Xubin Pan

**Affiliations:** ^1^ Institute of Plant Inspection and Quarantine Chinese Academy of Inspection and Quarantine Beijing 100176 China

**Keywords:** host range, plant defense, plant–insect interaction, phylogenetic signal

## Abstract

Insects tend to feed on related hosts. Coevolution tends to be dominated by interactions resulting from plant chemistry in defense strategies, and evolution of secondary metabolisms being in response to insect herbivory remains a classic explanation of coevolution. The present study examines whether evolutionary constraints existing in host associations of economically important fruit flies in the species‐rich tribe Dacini (Diptera: Tephritidae) and to what extent these species have evolved specialized dietary patterns. We found a strong effect of host phylogeny on associations on the 37 fruit flies tested, although the fruit fly species feeding on ripe commercially grown fruits that lost the toxic compounds after long‐term domestication are mostly polyphagous. We assessed the phylogenetic signal of host breadth across the fruit fly species, showing that the results were substantially different depending on partition levels. Further, we mapped main host family associations onto the fruit fly phylogeny and Cucurbitaceae has been inferred as the most likely ancestral host family for Dacini based on ancestral state reconstruction.

## Graphical abstract

The present study examines whether evolutionary constraints existing in host associations of economically important fruit flies in the species‐rich tribe Dacini (Diptera: Tephritidae) and to what extent these species have evolved specialized dietary patterns. We found a strong effect of host phylogeny on associations on the 37 fruit flies tested, although the fruit fly species feeding on ripe commercially grown fruits that lost the toxic compounds after long domestication are mostly polyphagous. Further, we discussed the role of secondary metabolisms in feeding preference, and other suites of traits in the specialization of fruit fly dietary patterns.

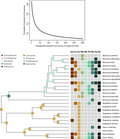

## 1 INTRODUCTION

Interactions between herbivores and host plants are thought to have contributed to speciation that might drive patterns of diversification (Ehrlich & Raven, 1964; Futuyma & Agrawal, 2009; Mitter et al., 1988). Most phytophagous insects tend to feed on a fraction of related plants, and host shifts are more common among closely related plant lineages and clades (Futuyma & McCafferty, 1990; Jaenike, 1990; Winkler & Mitter, 2008). The specialization of plant–herbivore interaction is of considerable relevance to the understanding of diversification and the extent to which plant–insect associations are specialized is key to understand the processes maintaining the diversity of both plants and insect herbivores. Phylogenetic information is embedded in the ways that insect herbivores interact with hosts; hence, phylogenetic approaches provide a historical framework to quantify dietary patterns throughout ecological and evolutionary processes. Accordingly, the level of specialization described by phylogenetic inference could be seen as an integrated measure of phenotypic and ecological attributes to detect specialization and evolution of host shifts (Gilbert & Webb, 2007; Rasmann & Agrawal, 2011).

Theory predicted that diversification of herbivore communities would be related to adaptation to plant defenses in a predictable manner (Farrell et al., 1991; Futuyma, 1983). The plant defense refers to a complex array of resistance traits and defensive function (Agrawal & Fishbein, 2006). Much of defense has been described with plant secondary chemistry which is viewed as the primary line of reducing herbivory, and it has been assumed as the determinant to shape specialized host associations and maintain relatively narrow host range (Agrawal & Weber, 2015; Dethier, 1941; Fraenkel, 1959; Macel et al., 2010; Zangerl & Berenbaum, 1993). Previous analyses showed that toxic secondary metabolites predicted host use in the red milkweed beetle (Rasmann & Agrawal, 2011). However, the specialization was also hypothesized to have resulted from morphological, physiological, and life‐history traits that functionally cause resistance against insects (Agrawal, 2007; Futuyma & Agrawal, 2009). Despite the evolutionary processes and ecological mechanisms shaping host utilization patterns are highly diverse, the explanation for specialization has been usually arising from the interplay between host phylogeny and plant defense.

Interacted hosts have always played a profound role on insect herbivores over the long‐term evolutionary history, and high diversity of insects has assumed to be attributed to their diverse interactions with plants. A number of studies that explore evolutionary scenarios of host utilization have been conducted on several genera of butterflies (Hardy, 2017; Janz et al., 2001), beetles (Rasmann & Agrawal, 2011), leaf mining moths (Lopez‐Vaamonde et al., 2003), and bees (Dellicour et al., 2014; Sipes & Tepedino, 2005). However, host use patterns on a variety of insect groups should be investigated for a comprehensive understanding of plant–insect interactions. Fruit flies (Diptera: Tephritidae), which constitute one of the most speciose families among phytophagous insects with nearly 5,000 described species, could be suitable candidates. Some species have caused the majority of direct economic impact in a wide range of fleshy fruits and vegetables and thus have received considerable attention in quarantine and international trade (White & Elson‐Harris, 1992). Additionally, members of Tephritidae are reported to form associations with various hosts that have exerted observable influences on fruit fly, such interactions present a highly complex phenomenon (Aluja & Mangan, 2008; Leblanc et al., 2012). Their associations with host plants have been well recorded as well. Comprehensive analysis on fruit fly dietary patterns from an evolutionary perspective is surprisingly scarce, our knowledge of evolution of host shifts in fruit flies which may contribute to a deeper insight into reciprocally interaction remains scant.

The tribe Dacini which forms a major part of the tropical and subtropical Tephritidae with over 900 currently described species (Doorenweerd et al., 2018). Ten percent of currently recognized species within Dacini are serious pests that infest a broad range of economically significant fruits and vegetables (Doorenweerd et al., 2018). Commercial hosts have lost the toxic compounds after long‐term domestication and artificial selection, toxic chemicals are thought of absence in ripe and overripe fruits that most fruit flies preferentially attack (Aluja & Mangan, 2008; Meyer et al., 2012). Given the high proportion of polyphagous species within the tribe Dacini, some were hypothesized to overcome plant defenses with little or no fitness cost, making fruit fly host expansion more easily achievable even not within a phylogenetic context (Clarke, 2017). This hypothesis was also linked with the effect of secondary metabolisms. Due to relatively high degrees of polyphagy, the dietary patterns of Dacini species have been much debated, and thus, species‐level analyses on host utilization of Dacini fruit flies within a phylogenetic framework are needed.

Here, we analyze the evolution of host associations in a species‐rich taxon of fruit flies. This study had four main goals: (a) to compile host association data for Dacini fruit flies, and obtain phylogenetic tree with branch length for host species; (b) to test phylogenetic signal in fruit fly host breadth data; (c) to investigate the role of host phylogeny in shaping fruit fly dietary patterns in a quantitative manner; (d) to reconstruct ancestral host states in the Dacini on a time‐calibrated phylogeny.

## 2 METHODS

### 2.1 Study group

**FIGURE 1:**
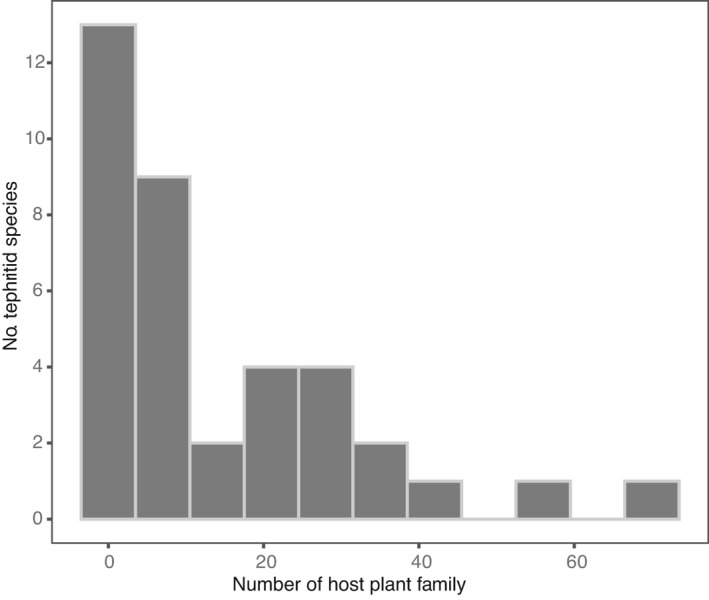
Frequency distribution of the number of host plant family

The tribe Dacini, primarily comprises species of three genera (*Bactrocera* Macquart, *Dacus* Fabricius, and *Zeugodacus* Hendel, is a species‐rich radiation which has been assumingly associated with diverse feeding patterns (De Meyer et al., 2015; Virgilio et al., 2009; Krosch et al., 2012). The taxonomy of Dacini has been getting much attention for decades, and recently, an overview of checklist for Dacini fruit flies became available (Doorenweerd et al., 2018). For the present study, we selected a total of 37 Dacini fruit flies that are foraging exclusively on fruit and flower structures of commercial plants, the taxonomy closely followed the species list suggested by the checklist. These species vary from monophagous (feeding exclusively on a single plant species) to polyphagous (feeding on plants of many families), 8 of the 37 fruit flies (21.62%) are restricted to a single host family, and 11 of the 37 species (29.73%) are feeding on 20 or more plant families (Figure 1); they consist of genera *Bactrocera* (24 spp.), *Dacus* (4 spp.), and *Zeugodacus* (9 spp.). To obtain the interacted hosts of the tephritid species, we extracted records from the pest‐oriented database of the Centre for Agricultural and Biosciences (CABI, https://www.cabi.org/cpc) which reports fruit flies occurring and feeding on plants. Host associations were cross‐checked for completeness with the COFFHI database (The Compendium of Fruit Fly Host Information, Edition 5.0 [https://coffhi.cphst.org/]; Liquido et al., 2019). Then, we held information from publications about field observation and infection studies to complement. On this basis, we excluded cases that were considered accidental occurrences and doubtful records in literature. Host records were designated as invalid if not identified to the species level and not included in the present study. Host families follow the plant family nomenclature outlined in the Angiosperm Phylogeny Group classification (Stevens, 2001). Accepted names, spellings, and taxonomy for host plants were standardized according to The Plant List (TPL, https://www.theplantlist.org/). Synonyms on the host list were eliminated and only accepted names adopted for each plant species. In general, our database of interactions between fruit flies and host plants contains 1,841 associations, including 37 Dacini fruit flies and 706 host species belonging to 87 families.

### 2.2 Phylogenetic analyses

Due to a great concern for taxonomy and diversification of Dacini species, the tribe Dacini has been extensively studied in view of phylogenetic understanding, and several phylogeny estimations are available (Krosch et al., 2012; Muraji & Nakahara, 2001; San Jose et al., 2018; Virgilio et al., 2009, 2015). The phylogenetic inference for Dacini used in the present study was taken from a well‐resolved phylogeny that is constructed on seven different gene regions (one mitochondrial and six nuclear genes) by San Jose et al. (2018), which comprises a comprehensive sampling of genes and represents all major groups within the tribe. It enables the evolutionary reconstruction of host associations in the tribe on a global scale.

**FIGURE 2:**
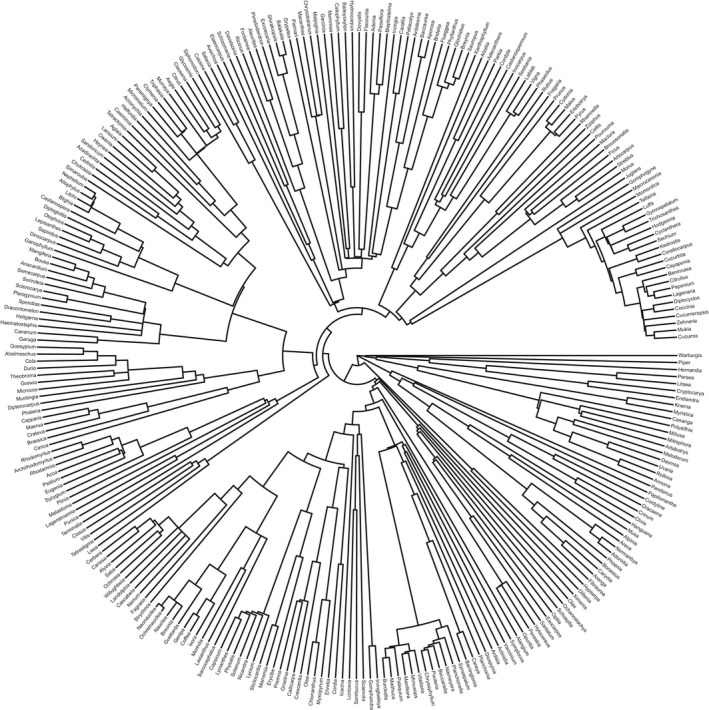
Phylogeny for dataset of the 286 host genera included in the analyses

The phylogenetic hypothesis for the set of included angiosperms was based on the most up‐dated mega tree for seed plants (Smith & Brown, 2018). We used the function phylo.maker in the R package “V. PhyloMaker” (Jin & Qian, 2019) coupled with Scenario 3 to bind species onto the tip of the backbone tree and then reconstructed a phylogeny for host plants with branch length. The species‐level phylogeny reconstruction which has been available for large numbers of plant species is demonstrated to be an efficient tool. Despite the uncertainty such as polytomies, the method that has been applied to explore phylogenetic relationships contributes to achieve further ecological and evolutionary insights. Although we have assembled the records at the species level and the phylogeny for host plant species was available, we investigated host patterns at the levels of genus and family in the analyses for which offers a more reliable and informative source (Figure 2).

### 2.3 Phylogenetic conservatism evaluating

To test whether host breadth and namely states of polyphagy would be phylogenetically distributed across the tephritid species tree, we used Blomberg's *K* (Blomberg et al., 2003) and Pagel's *λ* (Pagel, 1999) to assess the phylogenetic signal. We considered host breadth (i.e., the number of host families used by fruit flies) (a) as a continuous variable describing host plant pattern, which seems biologically appropriate, and (b) as binary variables, wherein we considered the state of polyphagy at various levels. Given that most economically significant fruit flies within Dacini are polyphagous, we defined “polyphagous” fruit flies as species that feed on more than two host families, three host families, and four host families, respectively. We investigated the phylogenetic signal in these characters, that is whether closely related fruit fly species are more likely to share a character state than expected from chance. Blomberg's *K* compares the observed distribution of tip data to expectations derived from a Brownian motion (BM) model of evolution, values of *K* > 1 imply strong phylogenetic signals, and *K* = 0 implies the absence of phylogenetic conservatism. While there is no clearly defined *K* value cutoff in which to apply phylogenetic comparative methods, nonsignificant value of <1, or more conservative <0.5, are typical for characters that are phylogenetic independent. Pagel's *λ* examines the effect of ancestral relatedness on character evolution, with a value close to 1 indicates phylogenetic signal and a value approaching 0 indicates phylogenetic independence.

We took a logistic regression approach to calculate the probability of a host plant being infected by, or found in association with, a particular tephritid species (or all of the tephritid species), and its relationship with phylogenetic distances. We compiled associations at the genus level, and the associations were modeled as a binary trait (0 = no interaction, 1 = interaction). In the regression analysis, we only considered fruit fly species with three or more plant associations to make it more reasonable, and host phylogenetic distances were calculated in million years. We integrated the associations between fruit flies and host plants with the matrix of pairwise phylogenetic distances among host genera to investigate the role of host phylogeny. To calculate the possibility of fruit fly host affiliation, we applied the phylogenetic distance as predictor variable [transformed as log_10_ (phylodistance + 1) according to empirical experiments from Gilbert et al. (2007, 2012)], and the responsible variable was 1 (infected by fruit flies) or 0 (assumed to be resistant to fruit flies). Generally, for each fruit fly, one interacting host was randomly selected as source host, and then, each of the other hosts was selected iteratively at random. The procedure was repeated for 1,000 total runs, with new random selections of source host for each fruit fly. The intercept (*β*_0_) and slope coefficient (*β*_1_) and the 95% confidence interval were obtained from the mean coefficients across all 1,000 sets from these regressions for all fruit flies.

### 2.4 Ancestral host state estimation

We applied the DEC (dispersal–extinction–cladogenesis) model (Ree & Smith, 2008) to infer ancestral host states for Dacini using R package “BioGeoBEARS” (Matzke, 2013). A DEC model assigns probability to various range‐changing events, and these event probabilities are used to calculate the likelihood of states based on maximum‐likelihood (ML) approach. DEC models are well suited for estimating phylogenetic histories of multistate discrete character such as geographic or host range and were initially applied on the biogeographical studies. Nevertheless, DEC models have done a better job of estimating phylogenetic evolution of diet breadth and vastly outperformed independent reconstruction of host use traits (Hardy, 2017). Specifically, we used the DEC* model in which the ancestral null states are disallowed and no stratification was applied (Massana et al., 2015).

First, to rescale branch length to time and create a time‐calibrated tree for Dacini, we fitted the phylogeny for a chronogram under the strict clock model in which evolutionary rates are assumed to be equal among branches. We used penalized likelihood approach to estimate by function chronos in R package “ape” (Paradis, 2013; Paradis et al., 2004). To reconstruct ancestral host states for Dacini, we estimated host evolution using main host taxa which are the most commonly used nine plant families among all associations of 37 fruit flies. This required dropping the fruit fly *Bactrocera olea* which was oligophagous on *Olea* spp. within the family Oleaceae. We classified 25 of the 37 fruit fly species selected in the analysis; only fruit flies feeding on less than 20 host families were remained. We excluded extremely polyphagous species as dietary pattern for these fruit flies might be outside their ancestral host range through abiotic factors such as long‐distance dispersal events. Finally, we mapped main dietary patterns of these fruit flies onto the phylogeny to estimate ancestral host states for Dacini species.

All statistical analyses were performed in R 3.6.3 (R Core Team, 2020).

## 3 RESULTS

We found that the phylogenetic signal of host breadth in Dacini fruit flies depended on the partition levels (i.e., polyphagy state). The character (feeding on) “three families” showed strong phylogenetic signals using both the *K* estimate and the *λ* estimate, whereas the continuous variable did not. The phylogenetic signals in the characters (feeding on) “two families” and “four families” were not apparent using the *K* estimate, but signals were detected under the *λ* estimate (Table 1). Significant values in the character “three families” indicated that these “polyphagy” clades (fruit flies feeding on more than three host families) might be generally conservative; however, when classifying host breadth as continuous variable, it did not show any evolutionary constraints on diet breadth within these Dacini species.

**TABLE 1:** Parameter estimates for phylogenetic signal in host breadth data of fruit fly species, according to Blomberg's *K* and Pagel's *λ*

Character	No. of fruit fly species	Blomberg's *K*	*p*‐Value	Pagel's *λ*	*p*‐Value
Continuous		0.115	.885	0.163	.127
Two families	27	0.740	.**000**	1.000	.**000**
Three families	24	1.082	.**000**	1.000	.**000**
Four families	22	0.846	.**000**	1.000	.**000**

Notes

*p*‐Values were derived using 10,000 randomizations and estimates statistically significant with a *p*‐value of <.05 are indicated in bold. The characters are different partition levels or different definitions of polyphagy. Number of fruit fly species is the number of polyphagous species counted based on characters.

**FIGURE 3:**
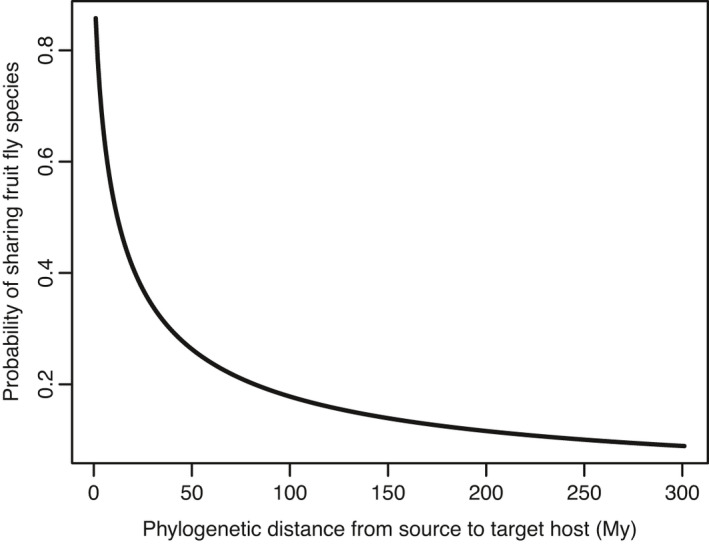
Phylogenetic test in the likelihood of host plant genera sharing fruit fly species. The slope coefficient for phylogenetic distance was significantly lower than zero, and 95% CI for β0 and β1 was 1.1883 to 2.4041 and –1.9242 to –1.4024, respectively

The probability that two host genera sharing one tephritid species declined significantly with phylogenetic distance, supporting that Dacini fruit flies prefer to feed on closely related plants (Figure 3). The logistic regression from empirical association testing of fruit fly species was logit(S) = 1.7962–1.6633*[log10(phylodistance + 1)]. The estimates showed host specialization in the fruit flies dietary pattern, with the decreasing probability of host sharing as a function of increased phylogenetic distances, that is the possibility of a fruit fly from a source host also attacking a target host. Nonetheless, the slope was relatively modest, showing that the phylogenetic relationships of host plants would be a weak predictor of host sharing of all related tephritid species. Besides, an equation of possibilities (Prob = exp(logit(S))/[1 + exp(logit(S))]) could be obtained by applying the regression coefficients to the logistic transformation of phylogenetic distances.

There were 87 families being used as hosts; however, only a handful of families are “important” host families which might play a decisive role in the evolutionary history of Dacini fruit flies. It contained nine main host families exclusively in the ancestral host state reconstruction analysis for studying purpose. Families of Cucurbitaceae and Myrtaceae were the most frequently used two families in terms of number of associated host plants belong to these families; families of Cucurbitaceae and Rutaceae were the most commonly used two families in terms of number of fruit fly species feeding upon these plant species. In addition, host use family Cucurbitaceae was distributed widely in the diet on genera of *Zeugodacus* and *Dacus*.

**FIGURE 4:**
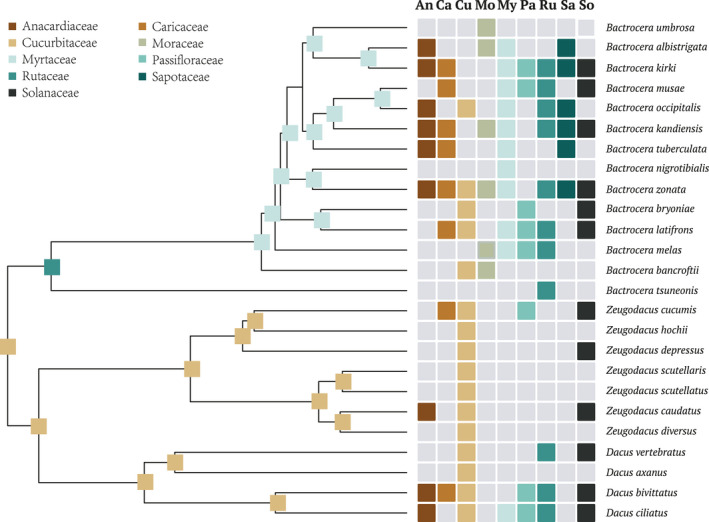
DEC* reconstruction of the use of host plant families over part of the fruit fly phylogeny. Ancestral host states for Dacini estimated using BioGeoBEARS, where tips represent main host distributions. Colors correspond to nine main host families: Anacardiaceae, Caricaceae, Cucurbitaceae, Moraceae, Myrtaceae, Passifloraceae, Rutaceae, Sapotaceae, and Solanaceae

Several extremely polyphagous species in the genus of *Bactrocera*, and two *Zeugodacus* species *Zeugodacus cucurbitae* and *Zeugodacus tau* were not included in the reconstruction, because that these fruit flies feeding on more than 20 host families seemed to be out of limitations due to shifts in geographic distribution and could do little to estimate ancestral hosts. There were 25 tephritid species that had been expected to be contributed to estimate the ancestral host state in the DEC model. We mapped main host families onto simplified phylogeny of tephritid species, most likely ancestral host states were shown on each node (Figure 4). In our results, Cucurbitaceae was reconstructed as the ancestral host in Dacini as a whole. The first split in the tribe Dacini coincided with host shift between the two clades, giving rise to genus *Bactrocera* in the host family Rutaceae and remaining Cucurbitaceae in genera *Zeugodacus* and *Dacus*. Cucurbitaceae was the most likely ancestral host for all nodes in fruit fly genera *Zeugodacus* and *Dacus*. The inferred ancestral host estimation for most nodes of genus *Bactrocera* referred to Myrtaceae except one node, for which Rutaceae was the most likely ancestral host family.

It is noteworthy that feeding preference for Cucurbitaceae was shared in common with most *Bactrocera*, *Dacus,* and *Zeugodacus* species. Many of *Zeugodacus* fruit flies are particularly known or suspected to breed in the fruits and flowers of Cucurbitaceae. However, host plants belonging to families of Moraceae and Sapotaceae, which had been used commonly in *Bactrocera* species, were absent in most species in the genera of *Dacus* and *Zeugodacus*. The exceptions were species of *Zeugodacus cucurbitae* and *Zeugodacus tau* which had been recognized as extremely polyphagous fruit flies and excluded in the reconstruction analyses. The *Bactrocrea* fruit flies hosts within the two families were restricted to species in the plant genera of *Artocarpus*, *Ficus,* and *Manilkara*.

## 4 DISCUSSION

Our analyses revealed patterns of host utilization in Dacini fruit flies and examined the effect of host plant phylogeny on the structure of the host plant–fruit fly associations. The constraints acting on host choice of polyphagous Dacini fruit flies display a similarity to those emerging from studies of plant–insect interactions, supporting that closely related insects are most likely to feed on closely related host plants. Further, we identified that the phylogenetic signal of diet breadth in Dacini might be critically dependent on the partition level at which polyphagy state is defined. Mapping main host taxa onto a fruit fly phylogeny indicates that the Cucurbitaceae has been reconstructed as the most likely ancestral host family of Dacini species.

It has been widely suggested that herbivores are artificially classified into categories “generalist” and “specialist” based on the degree of feeding specialization on the taxonomic level. Nonetheless, generalists may appear to be specialists owing to ecological or geographic restriction, whereas specialists may appear in the guise of generalists owing to their occurrence on a diverse but phylogenetically limited plant taxon. However, there has been a long‐standing hypothesis that specialist and generalist insects interact with plants in distinct ways. Generalist herbivores were assumed to use mechanisms to suppress plant defenses more than specialists, allowing them to feed on a broad range of species (Ali & Agrawal, 2012). Our results show that the degree of phylogenetic signal calculated varies with regard to how polyphagy is defined, suggesting that the way host breadth is measured would affect the result. The results provide the evidence that the classification of insect species according to diet breadth (the number of host plants used) may have a significant impact on phylogenetic inferences. We suggest that biological interpretations should be examined if different partitions of host breadth (e.g., specialist and generalist) are treated as opposite sides in future analyses.

We studied the effect of host phylogeny in the structure of plant–fruit fly associations, showing that the possibility of host sharing declines continuously as a function of phylogenetic distance. Generally, the more phenotypically and ecologically similar it is to the source host, the more suitable a plant it would be as a host. Traits governing the interactions between plants and insects may covary with phylogeny, this theory can explain the relationship between host shifts and phylogeny but remains to be proved. Relatively high levels of specialization were found always being linked to low number of host species or depending on the taxonomic diversity in the empirical analyses, which will cause overstatement of their significant in the specialization test. Despite high degrees of polyphagy, fruit fly species show feeding preferences over their distribution of hosts in the present study. Importantly, that we can reveal specialized host use patterns for Dacini fruit flies is especially notable. Dacini species, included in this study, are composed of fruit flies foraging exclusively on economically significant host plants. Indeed, host utilization of Dacini species may not be typically explained by secondary metabolites which have seemed to be the driver in most phytophagous insect dietary specialization patterns, this is because plant chemical defenses are expected to have been weakened during selective breeding and also the defenses are costly to produce (Rhoades, 1979).

Classic hypotheses predicted a strong correlation between coevolution and secondary metabolites, having exhibited nonrandom feeding patterns of phytophagous insects, as well as accounted for the ability to exploit a new host plant that its closely related relatives can attack (Ehrlich & Raven, 1964; Jaenike, 1990). In fact, the decisive role of secondary metabolites that seem to have been dominant in plant–insect interactions is far from absolute. Alternatively, increasing evidences suggested that there could have been a comprehensive suite of plant features involved in defenses against herbivores. A meta‐analysis concluded that plant secondary chemistry did not significantly predict resistance to insect herbivores and failed to detect any association between the concentrations of secondary metabolites and herbivore susceptibility (Carmona et al., 2011). Our result implies the phylogenetic conservatism of host use in tribe Dacini, and the secondary chemistry may have done little on specialized pattern of these economically significant fruit flies. Using such indirect evidence may be necessary, because disentangling the individual factor leading to an association between insects and hosts will not be achievable. It is unlikely to rule out the role of chemicals and link specific plant response to impacts on herbivores with much certainty.

The heritable variable in other suites of traits (i.e., life‐history, morphological, and physical resistance traits) that can evolve as adaptive syndromes may be relevant to plant–insect interactions. Although ripe, fleshy fruits function primarily to attract seed dispersers, they must also defend against diverse communities of frugivores. In particular, fruits can employ a variety of strategies such as ripening in seasons in which herbivores population densities are low or, reducing nutrient content and evolving a thick protective exocarp (i.e., physical defense). Alternatively, inducible volatile organic compounds involved in host recognition may function as indirect defenses (Cunningham et al., 2016; Rowen & Kaplan, 2016). For instance, *Rhagoletis pomonella* is a model for sympatric speciation (i.e., divergence without geographic isolation) by host shifts, that fruit odors as key traits help distinguish among respective host plants (Linn et al., 2003). And, other than host recognition, the evolution of olfactory mechanisms involved in host choice has an effect on fruit fly host range. Indeed, there are both visual and olfactory stimuli in the process of host recognition of female flies, which may lead a more complicated association between plants and fruit flies. The extent to which plant benefits from consumption of ripe fleshy fruits would affect trade‐offs presented by the investment in defense and attractant function. Fruit nutrient composition has also proved to be important factor influencing tephritid communities (Maud et al., 2017). Furthermore, much of the coevolution argument has centered on the evolution of plant traits, but it suggests that host shifts may be guided in part by limitations on genetic variation in insect species (Futuyma & Agrawal, 2009).

Host shifts have been shown to lead to variation in the relationships between host plants and fruit flies while adaption to feeding on different host species may have promoted sympatric speciation and diversity of tephritid species. Ancestral tephritids most probably evolving from a saprophagous to a phytophagous lifestyle have been considered as an example of opportunist (Aluja & Mangan, 2008; Wilson et al., 2012). The tribe Dacini contains several highly polyphagous tephritid species and some of those have invaded several countries, and long‐distance dispersal into new habitats might fascinating host broadening outside of their ancestral host. The ecological attributes and behavioral mechanisms adopted by these tephritids during their lifetime probably contribute to the highly polyphagous nature. Such characteristics may account for capabilities to become established in niches less utilized by other frugivorous tephritids, which could in turn maintain population by reproduction on a wide range of cultivated and wild host plants and consequently help facilitate its spread and invasiveness. Higher plant diversity may facilitate spillover across alternative host species. For Dacini species, we suggest that diverse host associations might be relevant to speciation events which cause extensive radiation. Earlier studies have confirmed that host phylogeny accounts for some of the structure of the fly community (Hafsi et al., 2016). To determine which traits underlie host associations and drive high diversification as a result of selective response to diverse host plants for Dacini is highly desirable.

Host status (i.e., a plant species could be infected or not) is an evolutionarily dynamic phenomenon and host breadth should be treated as a continuum (expand or shrink over evolutionary time), its evolution is thus particularly difficult to test due to the inherent uncertainties. It requires systems approaches that could be capable of handling the practical situation of preference and performance on a case‐by‐case basis especially for highly polyphagous species which exhibit a great degree of variability in their host use patterns. However, combining host utilization with information of bioinformatics and phylogenetic analyses is still a proven approach to investigate insect species dietary pattern and feeding strategy. Furthermore, although the association data included were cross‐checked, the identification of these fruit flies on hosts might actually be incorrect (i.e., sibling species pair with very limited morphological differentiation) or based upon accidentally incidence on putative hosts (i.e., some are likely to be either secondary infestations of fruit already infested by other species). The existence of complexes could also confuse fruit fly host affiliations. We assigned the host records at the level of host genus or family that could be more reliable and reasonable for the analyses, and dubious host records are omitted. Nonetheless, an extremely polyphagous fruit fly might consist of a complex of cryptic species, leading to overestimation of host breadth in the fruit flies. However, it means that the conservatism should be more evolutionarily constrained than we investigated.

## 5 CONCLUSIONS

We found that Dacini fruit flies feeding on economically important host plants which normally lost secondary metabolisms after long‐term domestication still show specialized host associations, implying a decrease in the possibility of host sharing with increasing phylogenetic distance of the host plant taxa. Earlier study proposed that polyphagous fruit flies should be able to infest a broad range of phylogenetically distant host plants without absolute limitations and overcome plant defenses with little effort. Our study disaccords with this view and suggests that host specialization cannot be explained solely by secondary metabolites. Cucurbitaceae was recognized as the most probably ancestral host family in ancestral host reconstruction analysis. Diverse associations outside ancestral hosts for extremely polyphagous fruit flies are potentially attributable to capabilities of long‐distance dispersal and being generalist opportunists.

## CONFLICT OF INTEREST

The authors declared no conflicts of interest.

## AUTHOR CONTRIBUTION

**Jiayao He:** Conceptualization (equal); Data curation (supporting); Funding acquisition (equal); Methodology (supporting); Visualization (supporting); Writing‐original draft (supporting); Writing‐review & editing (lead). **Ke Chen:** Conceptualization (equal); Funding acquisition (supporting); Project administration (equal); Supervision (equal); Validation (equal); Visualization (equal). **Fan Jiang:** Conceptualization (equal); Funding acquisition (supporting); Investigation (equal); Validation (lead); Writing‐review & editing (equal). **Xubin Pan:** Conceptualization (equal); Investigation (equal); Project administration (equal); Supervision (lead); Writing‐review & editing (equal).

## Supporting information

Data S1
